# Generative AI-assisted clinical interviewing of mental health

**DOI:** 10.1038/s41598-025-13429-x

**Published:** 2025-10-29

**Authors:** Sverker Sikström, Rebecca Astrid Boehme, Mariam Mirström, Thibaud Agbotsoka, Gergő Győri, Marta Lasota, Mona Tabesh, Lotta Stille, Danilo Garcia

**Affiliations:** 1 Department of Psychology, Lund University, Lund, Sweden; 2 Promotion of Health and Innovation for Well-Being (PHI-WELL) Lab, University of Stavanger, Stavanger, Norway; 3 Centre of Functionally Integrative Neuroscience, Aarhus University, Aarhus, Denmark; 4 Center for Research on Personality Development, SWPS University, Poznan, Poland; 5 Università degli Studi di Milano-Bicocca, Milan, Italy; 6 Department of Social Studies, University of Stavanger, Stavanger, Norway; 7 Department of Behavioral Sciences and Learning, Linköping University, Linköping, Sweden; 8 Lab for Biopsychosocial Personality Research (BPS-PR), International Network for Well-Being, Linköping, Sweden; 9 Centre for Ethics, Law and Mental Health (CELAM), University of Gothenburg, Gothenburg, Sweden; 10 Department of Psychology, University of Gothenburg, Gothenburg, Sweden

**Keywords:** AI-powered clinical interviews, Mental health assessment, Large language models (LLMs), Generative AI, Major depressive disorder (MDD), Generalized anxiety disorder (GAD), Obsessive-compulsive disorder (OCD), Post-traumatic stress disorder (PTSD), Attention-deficit/hyperactivity disorder (ADD/ADHD), Autism spectrum disorder (ASD), Eating disorders, Substance use disorder (SUD), Bipolar disorder (BD), Information technology, Diagnosis

## Abstract

The standard assessment of mental health typically involves clinical interviews conducted by highly trained clinicians. While effective, this approach faces substantial limitations, including high costs, high clinician workload, variability in expertise, and a lack of standardization. Recent progress in large language models (LLMs) offer a promising avenue to address these limitations by simulating clinician-administered interviews through AI-powered systems. However, few studies have rigorously validated such tools. In this study, we used TalkToAlba to develop and evaluat an AI assistant designed to conduct clinical interviews aligned with DSM-5 criteria. Participants (*N* = 303) included individuals with self-reported clinician-diagnosed mental health disorders, namely, major depressive disorder (MDD), generalized anxiety disorder (GAD), obsessive-compulsive disorder (OCD), post-traumatic stress disorder (PTSD), attention-deficit/hyperactivity disorder (ADD/ADHD), autism spectrum disorder (ASD), eating disorders (ED), substance use disorder (SUD), and bipolar disorder (BD)—alongside healthy controls. The AI assistant conducted diagnostic interviews and assessed the likelihood of each disorder, while another AI system analyzed interview transcripts to verify diagnostic criteria and generate comprehensive justifications for its conclusions. The results showed that the AI-powered clinical interview achieved higher agreement (i.e., Cohen’s Kappa), sensitivity, and specificity in identifying self-reported, clinician-diagnosed disorders compared to established rating scales. It also exhibited significantly lower co-dependencies between diagnostic categories. Additionally, most participants rated the AI-powered interview as highly empathic, relevant, understanding, and supportive. These findings suggest that AI-powered clinical interviews can serve as accurate, standardized, and person-centered tools for assessing common mental disorders. Their scalability, low cost, and positive user experience position them as a valuable complement to traditional diagnostic methods, with potential for widespread application in mental health care delivery.

**Supplementary Information:**

The online version contains supplementary material available at 10.1038/s41598-025-13429-x.

## Introduction

Accurate diagnosis and assessment of mental health disorders pose critical obstacles in modern psychiatry. Existing methods, including self-reported questionnaires and semi-structured clinical interviews, are the foundation of diagnostic processes but come with notable limitations. Self-reported questionnaires, while efficient and widely used, are vulnerable to response biases, including social desirability and defensiveness, which can compromise their validity and reliability. Similarly, semi-structured interviews, such as the Structured Clinical Interview for DSM Disorders (SCID), offer detailed insights into psychopathological conditions, requiring significant time and expertise to administer^1^. These challenges are further aggravated by a global shortage of mental health professionals, creating an urgent need for innovative, scalable, and efficient diagnostic practices^2,3^.

Although clinical interviews allow for direct interaction, enabling clarification and observation, they are not without flaws. These interviews are susceptible to bias stemming from interviewer variability, inconsistencies in administration, and subjective interpretations^1,4^. Such limitations, combined with the demand for highly trained professionals to conduct these assessments, highlight the need for more standardized, but still personalized, accessible solutions. Recent advances in natural language processing (NLP) and artificial intelligence (AI) have paved the way for transformative tools that address some of these gaps. These technologies offer scalable, efficient, and objective approaches to mental health diagnostics that hold the potential to overcome the constraints of traditional methods^2,5^.

Recent studies have demonstrated the feasibility of AI tools in understanding human language and context, yielding accurate clinical assessments. For instance, chatbot-based evaluations for conditions such as depression and anxiety have shown that AI-driven systems can effectively replicate key elements of clinician-led interviews^4^. By implementing advances in large language models (LLMs) like GPT-4, these tools can analyze open-ended patient responses, producing diagnostic insights comparable to traditional methods. Moreover, AI applications reduce survey fatigue, improve diagnostic accuracy, and maintain patient engagement, thus addressing critical gaps in current diagnostic practices^6^. As a result, AI tools are emerging as vital resources for extending mental health care to underserved populations, where traditional methods are inaccessible^4,5^.

Despite these promising developments, the application of generative AI in clinical interviews remains underexplored. Traditional interviews are often regarded as the gold standard due to their comprehensiveness and adaptability. However, they are resource-intensive and susceptible to human limitations, such as subjective interpretations and inconsistent application. AI models trained on interview-based datasets have demonstrated high accuracy in predicting clinical outcomes, presenting a viable alternative or complement to clinician-administered assessments^6^. Notably, chatbot systems and conversational AI have been recognized for their ability to interpret nuanced human behaviors while providing empathetic, patient-centred interactions. This positions AI-powered clinical interviews as a compelling solution to the challenges posed by conventional interview methods, particularly in resource-limited settings^4^. In mental health settings, these capabilities have been tested through preliminary studies on AI-driven interventions for depression, anxiety, and stress, demonstrating moderate to high concordance with clinician assessments^7^. This suggests that generative AI could help mitigate the shortage of mental health professionals by providing cost-effective, on-demand screening and support.

However, the rapid progression of these models also raises vital ethical, clinical, and data privacy considerations. For instance, ensuring the confidentiality of sensitive patient data requires robust encryption and strict adherence to regulatory frameworks like HIPAA or GDPR^8^. Moreover, while generative AI can approximate human-like empathy in conversation, it remains susceptible to biases inherent in its training data, potentially magnifying health disparities if not carefully audited^7^. Consequently, many experts advocate a hybrid approach, wherein clinicians supervise AI-driven assessments to validate findings and maintain high diagnostic standards. Despite these limitations, the scalability and adaptability of generative AI present a compelling case for exploring its role in integrated mental health care systems, where it may serve as a crucial complement to human-led interventions rather than a standalone replacement.

This study aims to evaluate whether an AI-powered clinical interview can accurately assess and discriminate between common mental health disorders, as well as the extent to which patients experience the interview as person-centered and supportive. This evaluation is especially important given the potential advantages of AI-powered interviews, including scalability, efficiency, accessibility, and the ability to deliver consistent, person-centered care in clinical settings. By integrating NLP and LLMs, the AI system is designed to simulate clinician-administered interviews, comprehensively assess diagnostic criteria, and provide transparent justifications for its conclusions. We hypothesize (H1) that a single AI-powered interview will demonstrate validity equal to or greater than state-of-the-art rating scales in assessing the nine most common mental health disorders, with diagnostic accuracy evaluated against patients’ self-reported clinician diagnosis. Furthermore, we hypothesize (H2) that assessments derived from the AI-powered interview exhibit fewer co-dependencies among disorders compared to conventional rating scales. Finally, we hypothesize (H3) that patients will rate the AI-powered clinical interview as empathic, relevant, understanding, and supportive of their concerns.

To test our hypotheses, we recruited participants with self-reported, clinician-diagnosed cases of the nine most common mental health disorders, along with a group of healthy controls. A large language model (LLM) was then prompted to conduct a clinical assessment of each participants’ mental health. This AI-powered assessment was compared with standardized diagnostic rating scales specific to each disorder. The AI assistant was instructed to begin with open-ended questions to formulate a preliminary diagnostic hypothesis and then evaluate whether DSM-5 criteria were met. Finally, it provided a likelihood estimate for each of the nine disorders.

By evaluating the validity of AI-powered clinical interviews and their capacity to provide a positive, person-centered user experience, this study offers evidence that such tools may help address key limitations in current diagnostic practices, such as, scalability, standardization, and accessibility. In doing so, it contributes to the growing body of research on AI as a complementary innovation in the evolving landscape of mental health care.

## Method

### Design

This study builds upon data collected in^9^, where participants with or without mental health diagnoses completed standardized rating scales dedicated to measure specific mental disorders^9^. developed and collected open-response questions from the participants about their symptoms and related experiences. However, those responses were not used in this study. Participants who voluntarily re-enrolled from the prior study subsequently engaged in a clinical interview conducted via chat with an AI assistant for mental health assessment and diagnostic evaluation. Following the interview, participants rated their experience of the AI-powered assessment. Separately, another AI assistant analyzed the interview data to provide a summary, including the most likely diagnosis and justifications, which participants did not access.

### Participants

Participants for the current study were recruited through Prolific, an online research platform, as part of^9^ and included 550 participants, where 450 individuals have self-reported, clinician-diagnosed mental health conditions and 100 healthy controls. Each diagnostic group included 50 participants, encompassing major depressive disorder (MDD), generalized anxiety disorder (GAD), bipolar disorder (BD), obsessive-compulsive disorder (OCD), attention-deficit/hyperactivity disorder (ADHD/ADD), autism spectrum disorder (ASD), eating disorders (ED), substance use disorders (SUD), and post-traumatic stress disorder (PTSD). During the prescreening phase of the prior study, participants confirmed that they were diagnosed by a professional clinician and that the diagnosis is ongoing. Furthermore, they reported their treatment status and their diagnostic history. Participants were only included when they reported English as their first language.

For the current study, we recontacted participants. Resulting in a final sample size of 303 participants, comprising 248 individuals with mental health conditions and 55 healthy controls.

The final sample consisted of 170 female participants, 110 male, 20 non-binary, and 3 individuals who preferred not to disclose their gender identity, with a mean age of 40.0 years (SD = 12.1). Participants reported their highest education levels: high school (*N* = 122), undergraduate degree (*N* = 127), postgraduate degree (*N* = 45), or doctorate (*N* = 9). We explicitly included participants with comorbidities. This follows the rationale of ecological psychology, emphasizing the situational diversity of mental health as a dynamic interplay of psychological, emotional, environmental, and social factors. Participants were removed only when they failed at least one of the attention checks or provided nonsensical or low-quality text responses, which ensured a sufficient data quality.

### Ethics

The study was performed in accordance with relevant guidelines. Participants provided once again informed consent, tailored to the current study, ensuring their understanding of its purpose, procedures, and voluntary nature, with anonymity and GDPR compliance emphasized. The study was approved by the Swedish Ethical Review Authority (Etikprövningsmyndigheten; registration number 2024-00378-02).

### Measures

#### AI-powered interview

Participants completed an AI-conducted clinical interview using the TalkToAlba software platform (TalkToAlba.com; see Appendix B for sample dialog). The AI-powered interview can be used following permission from the first author. TalkToAlba is designed to support mental health professionals through various AI-assisted features, including an AI therapist delivering CBT, as well as tools for recording, transcribing, and analysing patient-clinician meetings and interactions. The TalkToAlba platform is currently used by clinicians across Sweden and other parts of Europe. For this study, participants accessed the interview via a secure web link and completed it in a web browser with internet access. They could choose to interact with the AI clinician either by typing and reading, or by speaking and listening. The AI system, powered by a LLM, responded to input within a few seconds, simulating a natural conversational pace.

The AI-powered interview was divided into three phases: a *hypothesis* phase, a *validation* phase, and a final *assessment* phase, the latter of which was not disclosed to participants. The full wording of the prompts used in each phase is provided in Appendix A.

The AI assistant was built using OpenAI’s GPT-4 architecture, specifically the “gpt-4-turbo-preview” configuration. No additional training to fine tune the model was applied, nor did we upload additional documents related to mental health assessments. The language model analyzed the full dialogue in real-time without automated annotations To keep consistency and reproducibility in responses, the model was initialized with a fixed seed value of 0 and a low temperature setting of 0.1; all other parameters followed OpenAI’s default configurations.

During the initial phase (i.e., hypothesis phase), the AI assistant engaged participants in a natural, conversational exchange aimed at exploring their mental health status. Through a series of open-ended questions, the AI assistant collected relevant information and formulated a preliminary hypothesis regarding the participant’s mental health condition. This hypothesis was grounded in the DSM-5 diagnostic framework and informed the next phase of the interview.

During the second phase (i.e., validation phase), the AI assistant conducted a structured, confirmatory clinical interview focused on validating the preliminary diagnosis. Drawing on DSM-5 criteria, the assistant assessed each diagnostic criterion one at a time, posing follow-up questions as needed to resolve uncertainties or ambiguities. This iterative questioning continued until all relevant criteria were addressed, and a clear and comprehensive diagnostic picture had emerged.

In the final and undisclosed phase (i.e., assessment phase), the AI assistant synthesized the information gathered to estimate the likelihood that the participant met criteria for each of the nine target mental health disorders. This assessment served as the AI-generated diagnostic output, which was later compared to standard rating scale results.

#### User experience evaluation

Following the interview, participants evaluated the AI assistant using both quantitative and qualitative measures. Using rating-scale questions, participants rated the AI on perceived empathy, relevance, understanding, and supportiveness. Participants also responded to open-ended questions, describing their experience in five descriptive words. Finally, participants indicated their preferences among different assessment modalities, comparing the AI-powered interview to traditional methods such as clinical-led interviews and traditional, standardized rating scales.

#### Rating scales of mental health disorders

Standardized rating scales were used to gather data on symptoms and to complement participants’ self-reported clinical diagnoses. These scales were completed by participants in Boehme et al. (in preparation) and used in this study to provide comparative data for AI assessments. For depression, we used the Patient Health Questionnaire-9 (PHQ-9)^10^, a nine-item tool using a 4-point Likert scale to measure depressive symptoms. Anxiety symptoms were assessed using the General Anxiety Disorder-7 Scale (GAD-7)^11^, which consists of seven items rated on a similar scale. Obsessive-Compulsive Disorder (OCD) was measured using the Brief Obsessive–Compulsive Scale (BOCS)^12^ ), consisting of 15 items rated on a 3-point Likert scale and one open-response item to categorize obsessions and compulsions. For bipolar disorder, the Mood Disorder Questionnaire (MDQ)^13^ was utilized, comprising 14 binary (Yes/No) items and an additional question rated on a 4-point Likert scale.

To screen for ADHD, Part A of the Adult ADHD Self-Report Scale (ASRS)^14^ was applied, consisting of six items rated on a 5-point Likert scale. Autism Spectrum Disorder (ASD) was assessed using the Ritvo Autism and Asperger Diagnostic Scale (RAADS-14)^15^, a 14-item tool using a 4-point Likert scale.

Eating disorders were assessed using the Eating Disorder Examination Questionnaire (EDE-QS)^16^, which comprises 12 items scored on a 4-point Likert scale. Substance abuse was measured using the Alcohol Use Disorder Identification Test (AUDIT)^17^, featuring eight questions on a 5-point Likert scale and two on a 3-point scale, and the Drug Abuse Screening Test (DUDIT)^18^, with nine 5-point Likert scale items and two 3-point items.

Finally, for Post-Traumatic Stress Disorder (PTSD), the National Stressful Events Survey PTSD Short Scale (NSESSS-PTSD)^19^ was implemented. This tool includes one open-text response for describing a traumatic event and nine items rated on a 5-point Likert scale.

Cut-off scores for binary categorization (i.e., presence vs. absence of a diagnosis) were based on established thresholds commonly reported in the literature: PHQ-9 ≥ 10 (depression), GAD-7 ≥ 10 (anxiety), BOCS ≥ 8 (obsessive-compulsive disorder), ASRS ≥ 10 (ADHD), NSE ≥ 14 (PTSD), RAADS ≥ 14 (autism spectrum), EDE ≥ 18 (eating disorder), DUDIT ≥ 25 (substance use), MDQ ≥ 7 (bipolar disorder).

#### Procedure

Participants provided written informed consent prior to participation, in accordance with approval from the Swedish Ethical Review Authority (Ref. No. 2024-00378-02). They then completed the AI-powered clinical interview, followed by a series of questions evaluating their experiences with the AI interaction during the AI-powered interview, their preferences regarding assessment methods, and self-reported demographic, and diagnostic information.

### AI assistant

An AI assistant, based on OpenAI’s GPT-4 architecture (gpt-4-turbo-preview) was created for assessing participants’ responses from the AI-powered clinical interviews according to the DSM-5 diagnostic criteria. The assistant was instructed to estimate the likelihood that each participant met the DSM-5 diagnostic criteria for the nine targeted disorders: major depressive disorder (MDD), generalized anxiety disorder (GAD), obsessive-compulsive disorder (OCD), bipolar disorder (BD), attention-deficit/hyperactivity disorder (ADHD/ADD), autism spectrum disorder (ASD), eating disorders (ED), substance use disorder (SUD), and post-traumatic stress disorder (PTSD). This AI-generated diagnostic measure is hereafter referred to as GPT. The exact wording of the prompt is provided in Appendix A. For binary classification purposes, a cut-off score of ≥ 50% likelihood was used to indicate presence of a diagnosis. That is, if the AI assistant estimated a probability of 50% or higher that the participant met the DSM-5 criteria for a given disorder, it was classified as present. This cut-off was chosen to reflect a neutral decision boundary, where the AI was at least as confident in the presence of the disorder as in its absence, aligning with conventional practices in probabilistic classification.

### Statistics

**Fig. 1:**
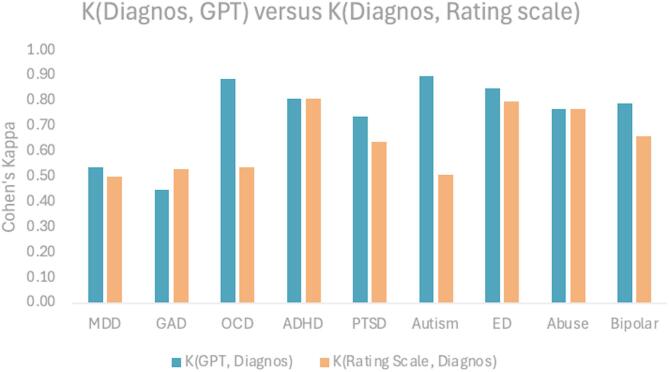
Cohen’s Kappa between diagnosis and GPT- and RS-based assessments. The figure shows Cohen’s Kappa between self-reported diagnosis made by a clinician and GPT-based assessment (in blue), as well as rating scales (RS) (in red) divided into the nine disorders.

Diagnostic assessments were validated against participants’ self-reported clinical diagnosis, which were based on prior assessments made by their treating clinicians (referred to as Diag.), using binary classification (Table 1; Fig. 1). Agreement between this reference outcome and the AI-generated diagnosis (GPT) as well as the standarized rating scales (RS) was evaluated using Cohen’s Kappa, which measures classification agreement beyond chance. In addition, t-tests were used to evaluate whether the proportion of agreement with self-report diagnosis (Diag.) differed between the GPT and RS.

## Results

### Frequency of occurrence

For most conditions, the number of self-reported diagnoses in the total dataset ranged between 32 and 57. However, notably higher numbers were reported for MDD (*N* = 193), GAD (*N* = 186), and PTSD (*N* = 98), indicating a high level of comorbidities (Table 1).

### Validation

 The results in Table 1 indicate no significant differences between the GPT and RS across all measures, except for eating disorder (ED); however, this effect disappeared following the Bonferroni correction of multiple comparisons. Thus, these data support hypothesis 1 (H1), the GPT has similar validity as RS in assessing common mental health disorders.

### Sensitivity and specificity

The sensitivity and specificity of the GPT and RS measures are found in Table 2. The sensitivity measures are higher for GPT and RS for all diagnoses except GAD and SUD. The specificity measures are higher for all GPT and RS diagnosis except for GAD, and it is equal for ADHD.

### Correlations

**Fig. 2:**
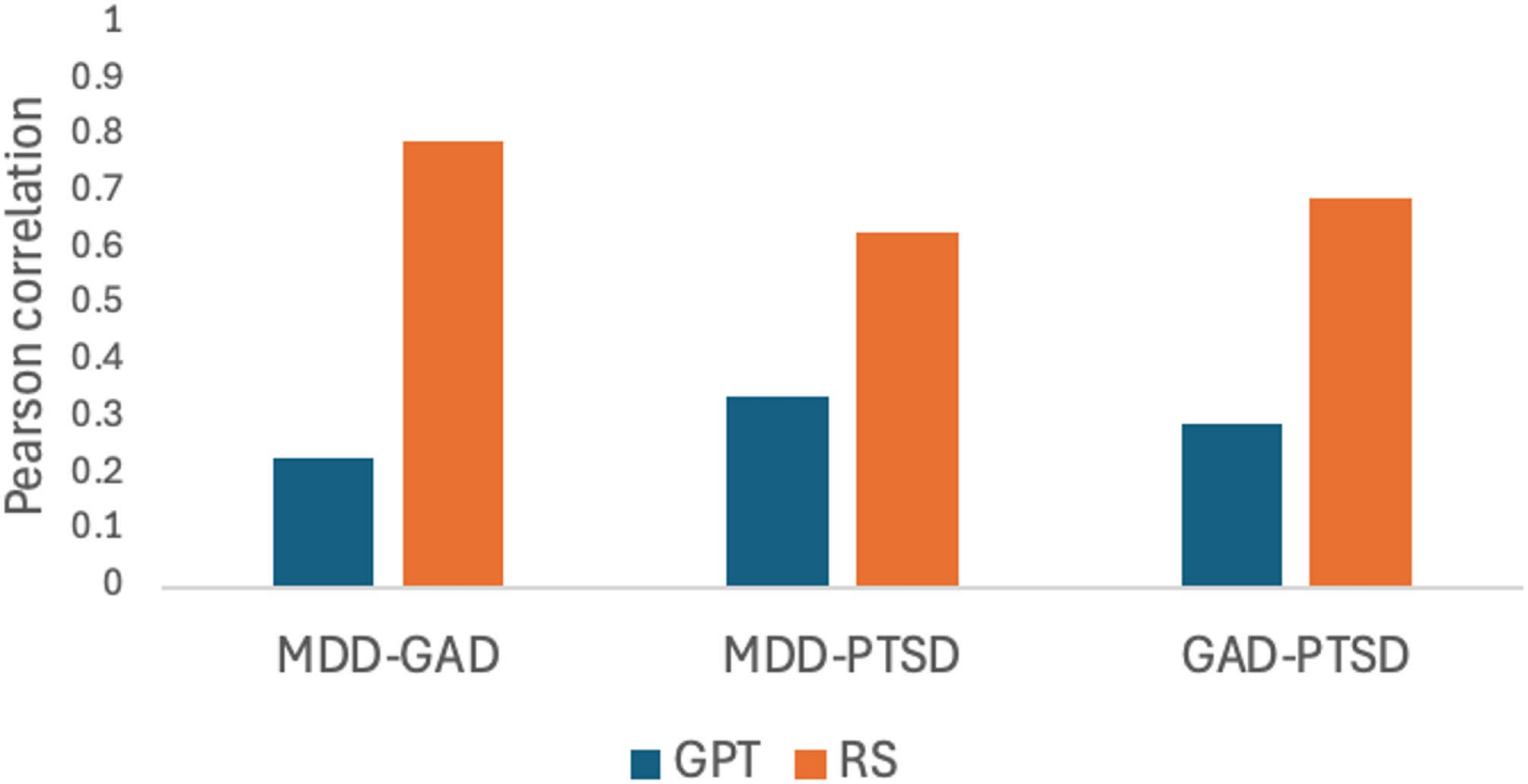
Pairwise Pearson correlation between MDD, GAD, and PTSD for GPT and RS assessments. The figure shows all pairwise Pearson correlations for the three most common disorders in the dataset (MDD, GAD, and PTSD) for GPT to GPT measures in blue and for RS to RS measures in red. All GPT-GPT correlations are significantly lower than the corresponding RS-RS correlations following Bonferroni corrections for multiple comparisons.

 The Pearson correlations between the GPT-GPT, RS-RS, and GPT-RS assessments for each pairwise diagnosis are found in Table 3. The GPT-GPT correlations (mean *r*-values = 0.25) were systematically lower than the corresponding RS-RS correlations (mean *r*-values = 0.43), where most of the differences were significant (except for correlations including ED or SUD). The pairwise correlations between GPT-GPT and RS-RS for the three most common disorders (MDD, GAD and PTSD) are shown in Fig. 2. Thus, these results support hypothesis 2 (H2), that GPT assessment has lower codependencies compared to RS.

### Patients’ experience

The participants rated the experience of chatting in the clinical interview as very, or extremely, empathic, relevant, understanding, or supportive in 57%, 72%, 65%, and 54% of the responses, respectively (Table 4). Thus, these results support hypothesis (H3) that GPT has lower co-dependencies compared to the RS.

**Fig. 3:**
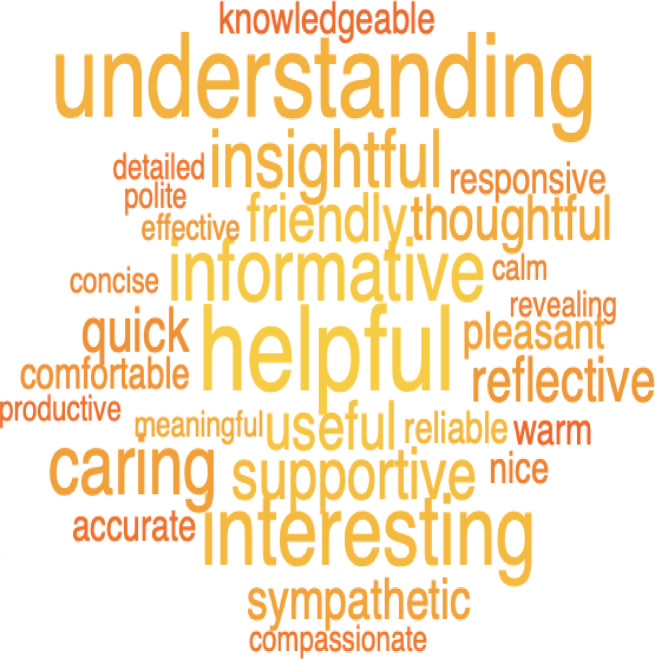
A word cloud of summarizing words generated by participants describing how they experienced the AI-supported clinical interview. The figure shows a word cloud summarizing the five words each participant generated to describe the experience of the AI-supported clinical interview. The word cloud was generated by plotting the 30 most significant words that discriminate generated words from a random subset of English words using a semantic t-test as described in^20^. The font size represents the frequency with which the participants generate the words, and the color indicates the significance levels (yellow being more significant than red).

Figure 3 shows a word cloud summarizing the five words each participant used to describe the experience of the AI-supported clinical interview. All the words in the cloud had a positive valence, as evaluated by the authors. The most common words were *understanding*, *helpful*, *interesting*, *informative*, and *caring*.

**Table 1:** Cohen’s kappa between assessments based on GPT, rating scales, and self-reported diagnosis.

Disorder	RS	GPT - Diag.	RS - Diag.	GPT - RS	*p*	*N*
MDD	PHQ9	0.54	0.50	0.46	0.0413	193
GAD	GAD7	0.45	0.53	0.48	0.5531	186
OCD	BOCS	0.89	0.54	0.48	0.0000	32
ADHD	ASRS	0.81	0.81	0.82	0.9157	57
PTSD	NSE	0.74	0.64	0.60	0.0866	98
ASD	RAADS	0.90	0.51	0.45	0.0000	31
ED	EDE-QS	0.85	0.80	0.81	0.1635	32
SUD	DUDIT	0.77	0.77	0.72	0.8789	31
BD	MDQ	0.79	0.66	0.57	0.0017	45

The table columns show the disorder, the rating scales (RS), the Cohen’s Kappa (K)between GPT-based assessment (GPT) and self-reported diagnosis (Diag), K between GPT and rating scale (RS), K between GPT and RS, significant testing based was based on a t-tests of agreement with self-reported diagnosis for GPT-Diag and RS-Diag (*p*), and the number of self-reported diagnoses (*N*).

**Table 2:** Sensitivity and specificity for the GPT and RS scales.

Disorder	Sensitivity GPT	Sensitivity RS	Specificity GPT	Specificity RS
MDD	0.74	0.66	0.45	0.43
GAD	0.63	0.66	0.39	0.47
OCD	0.89	0.57	0.88	0.52
ADHD	0.82	0.81	0.80	0.80
PTSD	0.75	0.69	0.72	0.60
ASD	0.90	0.54	0.90	0.49
ED	0.85	0.81	0.84	0.79
SUD	0.77	0.78	0.76	0.75
BD	0.79	0.68	0.78	0.64

The columns show the sensitivity and specificity for the GPT and rating scales (RS) metrics for each of the disorders in the rows.

**Table 3:** GPT-GPT, RS-RS, SR-SR, and GPT-RS correlations.

GPT-GPT	MDD	GAD	OCD	ADHD	PTSD	ASD	ED	SUD		Mean
GAD	** 0.23									
OCD	* 0.16	0.35								
ADHD	** 0.30	** 0.18	* 0.19							
PTSD	** 0.34	** 0.29	** 0.26	** 0.17						
ASD	** 0.14	** 0.15	** 0.25	0.48	** 0.21					
ED	0.54	** 0.17	* 0.21	0.19	0.29	0.13				
SUD	0.26	0.16	* 0.12	0.2	0.33	0.17	0.26			
BD	0.49	** 0.15	** 0.24	* 0.32	* 0.27	* 0.22	0.38	0.35		
*Mean*	*0.31*	*0.21*	*0.21*	*0.27*	*0.28*	*0.17*	*0.32*	*0.35*		*0.25*
RS-RS	MDD	GAD	OCD	ADHD	PTSD	ASD	ED	SUD		
GAD	0.79									
OCD	0.34	0.42								
ADHD	0.65	0.59	0.42							
PTSD	0.63	0.69	0.51	0.51						
ASD	0.48	0.48	0.50	0.52	0.48					
ED	0.45	0.42	0.38	0.30	0.42	0.25				
SUD	0.39	0.27	0.34	0.24	0.36	0.16	0.30			
BD	0.40	0.41	0.51	0.48	0.44	0.38	0.30	0.31		
*Mean*	*0.52*	*0.47*	*0.44*	*0.41*	*0.43*	*0.26*	*0.30*	*0.31*		*0.43*
SR-SR	MDD	GAD	OCD	ADHD	PTSD	Autism	ED	Abuse	Bipolar	
GAD	0.44									
OCD	0.08	0.14								
ADHD	0.19	0.23	0.14							
PTSD	0.29	0.36	0.18	0.20						
Autism	0.02	− 0.01	0.11	0.32	0.17					
ED	0.13	0.10	0.27	0.01	0.18	0.04				
Abuse	0.25	0.21	0.09	0.13	0.20	− 0.02	0.20			
Bipolar	0.05	0.14	0.16	0.06	0.23	0.05	0.22	0.15		
*Mean*	*0.18*	*0.17*	*0.16*	*0.14*	*0.20*	*0.02*	*0.21*	*0.15*		*0.16*
GPT-RS	MDD	GAD	OCD	ADHD	PTSD	ASD	ED	SUD	BD	
MDD	0.48	0.36	0.1	0.29	0.23	0.18	0.17	0.15	0.21	*0.24*
GAD	0.24	0.38	0.16	0.22	0.18	0.20	0.13	0.04	0.11	*0.18*
OCD	0.24	0.33	0.25	0.23	0.20	0.17	0.16	0.03	0.20	*0.20*
ADHD	0.21	0.18	0.11	0.35	0.20	0.16	0.03	0.02	0.24	*0.17*
PTSD	0.24	0.32	0.18	0.13	0.34	0.24	0.16	0.10	0.11	*0.20*
ASD	0.10	0.09	0.16	0.18	0.16	0.27	0.07	0.15	0.19	*0.15*
ED	0.49	0.40	0.23	0.25	0.34	0.21	0.32	0.22	0.24	*0.30*
SUD	0.20	0.23	0.20	0.19	0.20	0.20	0.25	0.09	0.14	*0.19*
BD	0.32	0.29	0.19	0.18	0.32	0.24	0.22	0.22	0.27	*0.25*
*Mean*	*0.26*	*0.28*	*0.19*	*0.22*	*0.26*	*0.21*	*0.17*	*0.11*	*0.19*	*0.21*

The table shows GPT-GPT (upper section), RS-RS (second section), SR-SR (third section), and GPT-RS (lower section) Pearson correlations for all pairwise diagnoses. Rows and columns labels show the mean correlations. The stars in the GPT-GPT section represent significantly lower correlations compared to the corresponding correlation in the SR-SR section, following Bonferroni correction (**) for multiple comparisons and without corrections (*).

**Table 4:** Patients’ experience of the AI-supported clinical interview.

Responses	Empathic	Relevant	Understanding	Supportive
Not at all	21	5	18	29
Slightly	61	29	40	62
Moderate	85	76	78	88
Very	158	164	155	138
Extremely	62	113	96	70
P (very + extremly)	0.57	0.72	0.65	0.54

The table shows the participants’ ratings of the extent they found the AI clinical interview to be *empathic*, *relevant*, *understanding*, and *supportive*. The first five rows show the number of participants indicating each response alternative, and the last row presents the likelihood that participants responded with *very* or *extremely*.

## Discussion

We aimed to investigate the potential of an AI-powered clinical interview, guided by DSM-5 criteria, to serve as a reliable diagnostic tool for common mental health disorders. Traditional diagnostic methods, including clinician-administered interviews, face significant limitations such as variability in expertise, high costs, and restricted scalability, particularly in resource-constrained settings^2,5^. By utilizing LLMs and NLP, we explored whether AI systems could match or exceed that diagnostic validity of state-of-the-art rating scales while improving accessibility, efficiency, and standardization.

Our findings support hypothesis 1 that AI-powered clinical interviews can achieve diagnostic validity that is comparable to, or exceeds, traditional rating scales for several common mental health conditions. The AI-generated assessments (GPT) showed higher agreement, as measured by Cohen’s Kappa, with self-reported clinician diagnosis for major depressive disorder, obsessive-compulsive disorder, autism spectrum disorder, and bipolar disorder. No significant differences were observed for generalized anxiety disorder, attention/hyperactivity disorder, post-traumatic stress disorder, eating disorders, or substance use disorder. Sensitivity and specificity measures followed a similar pattern, with GPT generally outperforming rating scales except in sensitivity for generalized anxiety disorder and substance use disorder. These results align with prior research highlighting the diagnostic potential of AI tools in replicating clinician reasoning and assessment accuracy^4,9^. While GPT showed generally high diagnostic agreement (Cohen’s Kappa > 0.70), major depression disorder and general anxiety disorder were exceptions. One possible explanation is that these disorders often co-occur as secondary diagnoses and may have been underrepresented in the AI’s assessment when another primary disorder dominated the interview. This reflects a common challenge in diagnostic assessment, accurately capturing comorbidities, particularly when symptoms overlap or are context-dependent. However, further studies are needed to investigate this hypothesis.

In this context, a well-known problem with rating scales of mental disorders is that they often have high correlations that do not always correspond to the actual comorbidity of the diagnosis. Notably, GPT outperformed rating scales in minimizing artificial co-dependencies between disorders, supporting Hypothesis 2. For example, the correlation between PHQ-9 (depression) and GAD-7 (anxiety) was *r* = .79, a level of association that likely overestimates true comorbidity since the correlation between the self-reported diagnosis was much lower (*r* = .44). In contrast, the GPT-generated scores for these same disorders showed a considerably lower correlation (*r* = .23), suggesting that the AI model could differentiate diagnostic constructs more effectively than traditional rating scales (i.e., hypothesis 2). This aligns with long-standing critiques of rating scales’ tendency to conflate overlapping symptom dimensions^21^ and with recent findings suggesting that traditional scales may lack the specificity required to distinguish closely related disorders in predictive modeling contexts^22^.

Our third hypothesis was that participants would perceive the AI-powered interview as *empathic*, *relevant*, *understanding*, and *supportive*. This was strongly supported by the data, participants rated the interview highly across all these dimensions, indicating that the system provided a positive, person-centered experience. These results align with emerging research showing that AI tools, when carefully prompted and structured, can simulate not only diagnostic reasoning but also emotionally attuned communication, person-centered interactions, and foster trust and engagement^5^. This suggests that LLMs, despite being non-human, are capable of delivering assessments that are experienced as respectful, attentive, and meaningful by users. These findings are especially important in light of longstanding concerns that AI systems may feel “cold” or too mechanistic, undermining the therapeutic alliance^23^. Instead, our results indicate that conversational AI can foster a sense of being heard and understood, even in highly sensitive contexts such as mental health evaluation. This opens the door to broader use of AI-powered assessments not only as diagnostic tools but as supportive interfaces that help patients articulate their experiences. Moreover, the consistent person-centered ratings across disorders and demographic groups suggest the approach may generalize well across clinical populations, although further research is needed to confirm this.

## Limitations

Despite the promising results, several limitations warrant consideration. First, the diagnostic validity of the AI-powered clinical interview was evaluated against participants’ self-reported clinician-diagnosed mental health conditions. Although these diagnoses were made by clinicians, the use of retrospective self-report introduces potential forrecall biases, misunderstandings, and the lack of control for variability in the quality of initial diagnoses^23^. A more robust design would involve validation against clinician-administered diagnostic interviews conducted in parallel with the AI assessment, such interviews are after all widely regarded as the gold standard in psychiatric assessment^1^. Furthermore, similar to clinical interviews with humans, we could not exclude the possibility that in a few cases participants spelled out the diagnosis directly to the AI-clinician, rather than the symptoms.

Second, while the AI-powered system was programmed to follow DSM-5 diagnostic criteria systematically, it lacked access to multimodal data, such as tone of voice, facial expression, and behavioral context; all of which human clinicians use to inform their judgement^1^. Prior research suggests that multimodal input (e.g., from video, audio, or biosensors) can improve diagnostic sensitivity and enhance the understanding of symptom expression, particularly in complex or comorbid cases^22^. This limits the AI’s capacity to detect subtle emotional or behavioral signals that may be clinically relevant.

Third, the performance of the AI system depends heavily on predefined models, prompt design, and user input. While prompt engineering, the process of structuring and refining input to optimize model responses, was carefully managed in this study, there is still a risk of variability or drift in how different users interact with the system or how LLMs respond under different conditions. This raises concerns about reproducibility and consistency in clinical applications unless model behavior is tightly controlled and monitored, which often require human judgment^24^ - For example. Complex or atypical symptom presentations may fall outside its structured response patterns, limiting its ability to fully capture nuanced information^2,25^. Unlike human clinicians, the AI lacks real-time adaptability to subtle verbal or behavioral cues, potentially leading to missed diagnostic insights.

The effectiveness of AI-powered clinical interviews hinges significantly on the careful design of prompts used to guide LLMs during patient interactions. Prompt engineering has indeed emerged as a pivotal component in tailoring systems for mental health diagnostics. Well-crafted prompts enable LLMs to navigate the nuanced linguistic and contextual aspects of clinical dialogue, ensuring that patient concerns are addressed empathetically, and diagnostically relevant information is captured. Recent studies have demonstrated that strategically designed prompts can enhance the model’s ability to adhere to diagnostic frameworks, such as the DSM-5 while mitigating the risks of irrelevant or biased outputs^6^. This alignment not only improves diagnostic accuracy but also reinforces the perceived empathy and supportiveness of AI interactions, which are critical for patient engagement and trust in mental health applications^4,26^.

Moreover, advanced prompt engineering techniques allow for dynamic and adaptive questioning, enabling the AI to adjust its approach based on patient responses. By integrating conditional logic and iterative refinement of prompts, LLMs can emulate clinician-administered diagnostic interviews with greater precision, addressing comorbidities and subtleties in patient narratives^5^. For instance, prompts that explicitly guide the model to ask follow-up questions or provide justifications for its diagnostic conclusions enhance the system’s interpretability and reliability^2^. These innovations make prompt engineering an indispensable tool for scaling AI-powered clinical interviews to diverse populations, particularly in resource-constrained settings. By ensuring that LLMs are both diagnostically rigorous and patient-centered, prompt engineering contributes to the broader goal of transforming mental health care through scalable, accessible, and standardized solutions.

Fourth, the study population may not represent the full diversity of mental health service users. Participants were recruited online and may have higher digital literacy or greater comfort engaging with AI systems than the general population. In addition, cultural and linguistic nuances were not systematically addressed, limiting the generalizability of the findings. These factors play a critical role in mental health presentations and interpretations, thus, future studies should include more diverse samples and evaluate whether the AI-powered interview performs equivalently across different demographic and cultural contexts^5^. Additionally, the recruitment method may have introduced selection bias, as participants were drawn from an online platform, potentially skewing the sample toward individuals with greater digital literacy or specific sociodemographic characteristics.

Finally, although the AI system was rated highly for empathy and supportiveness in this study, it remains uncertain whether such positive perceptions will persist in long-term or high-stakes clinical contexts. Evaluating the stability of user trust and engagement over time, across varying levels of clinical severity, and more emotionally charged situations will be essential for establishing the broader acceptability and ethical viability of AI-powered diagnostics. Continued assessment of user experience in diverse populations and use cases will be critical to ensuring these tools are perceived not only as accurate but also as trustworthy and supportive over time.

## Practical implications

The findings of this study underscore the transformative potential of AI-powered clinical interviews in addressing critical gaps in mental health care delivery. This tools offer a scalable, cost-effective, and standardized alternative that can alleviate the workload of overburdened clinicians and expand access to quality mental health assessments, particularly in resource-limited or underserved settings^2,5^. Notably, our results demonstrate that diagnostic precision can be achieved without sacrificing user experience, suggesting AI-powered clinical interviews can complement traditional clinical workflows while also offering additional benefits, including accessibility, standardization, low-cost alternatives, and enhanced patient experiences. Moreover, the system’s adherence to DSM-5 criteria secures consistency in diagnoses while providing the flexibility required in dynamic, real-world scenarios.

A key strength of the AI-powered interview is its ability to simulate empathetic, person-centered interactions. Participants rated the tool highly for empathy, understanding, and supportiveness, addressing a common critique that AI lacks human warmth. This positions AI systems as a viable complement to traditional methods, particularly for preliminary assessments or as part of telemedicine platforms^4^. Such tools could also help reduce stigma by delivering a private and judgment-free environment for individuals hesitant to seek in-person mental health care. As digital health ecosystems grow, such tools may be particularly valuable in telepsychiatry, mental health triage, or self-guided assessment pathways. Furthermore, the strict adherence to DSM-5 criteria ensures consistency across assessments and increases their potential for integration into broader public health systems. Governments, insurers, or healthcare organizations could deploy AI-powered assessments as scalable front-line tools to triage patients, accelerate referrals, and arrange timely intervention for individuals in need.

Future iterations of AI-powered interviews should continue to evolve by incorporating multimodal inputs, such as vocal tone, facial expression, sentiment analysis, and behavioral markers, which may further enhance diagnostic accuracy and personalization^9^. Equally important is the cultural and linguistic adaptation of AI-powered interviews to gurantee equity, relevance, and utility across global populations^23^. When these dimensions are taken into account, AI-powered interviewshave the potential to become an integral and inclusive element of modern mental health care.

## Conclusion

Our study demostrates the promise of AI-powered clinical interviews, powered by LLMs, as a reliable and scalable innovation in mental health diagnostics. The AI system achieved diagnostic accuracy that was comparable, and in some cases superior, to widely used rating scales across several common psychiatric disorders. Just as importantly, participants rated the AI-powered interviews as empathic, relevant, and supportive, challenging the notion that AI-based tools lack emotional resonance and person-centered sensitivity.

While this study relied on self-reported clinical diagnoses and excluded multimodal data, the overall findings support the integration of AI into current diagnostic workflows. In resource-limited or high-demand settings, AI-powered interviews may serve as accessible and standardized first-line assessments, enabling earlier intervention and more efficient use of clinical resources.With further refinement and development, including cultural and linguistic adaptation, and the integration of multimodal inputs, AI-powered clinical interviews could evolve into versatile tools that complement clinician judgment together with equity and scalability. Therefore, it revises global mental health care by increasing accessibility, efficiency, and precision. In addition, responsible implementation is a must to utilize this novel and innovative approach.

## Supplementary Information

Below is the link to the electronic supplementary material.

Supplementary Material 1

## Data Availability

The text data is not available as it includes personal information. Other data is available upon request from the corresponding authors.
